# DNA Condensation Triggered by the Synergistic Self-Assembly of Tetraphenylethylene-Viologen Aggregates and CT-DNA

**DOI:** 10.3389/fchem.2021.716771

**Published:** 2021-07-22

**Authors:** Sajena Kanangat Saraswathi, Varsha Karunakaran, Kaustabh Kumar Maiti, Joshy Joseph

**Affiliations:** ^1^ Photosciences and Photonics Section, CSIR-National Institute for Interdisciplinary Science and Technology, Thiruvananthapuram, India; ^2^ Academy of Scientific and Innovative Research (AcSIR), Ghaziabad, India

**Keywords:** DNA condensation, tetraphenylethylene, viologen, electrostatic interaction, self-assembly, aggregates

## Abstract

Development of small organic chromophores as DNA condensing agents, which explore supramolecular interactions and absorbance or fluorescence-based tracking of condensation and gene delivery processes, is in the initial stages. Herein, we report the synthesis and electrostatic/groove binding interaction–directed synergistic self-assembly of the aggregates of two viologen-functionalized tetraphenylethylene (**TPE-V**) molecules with CT-DNA and subsequent concentration-dependent DNA condensation process. **TPE-V** molecules differ in their chemical structure according to the number of viologen units. Photophysical and morphological studies have revealed the interaction of the aggregates of **TPE-V** in Tris buffer with CT-DNA, which transforms the fibrous network structure of CT-DNA to partially condensed beads-on-a-string-like arrangement with **TPE-V** aggregates as beads *via* electrostatic and groove binding interactions. Upon further increasing the concentration of **TPE-V**, the “beads-on-a-string”-type assembly of **TPE-V/CT-DNA** complex changes to completely condensed compact structures with 40–50 nm in diameter through the effective charge neutralization process. Enhancement in the melting temperature of CT-DNA, quenching of the fluorescence emission of ethidium bromide/CT-DNA complex, and the formation of induced CD signal in the presence of **TPE-V** molecules support the observed morphological changes and thereby verify the DNA condensation abilities of **TPE-V** molecules. Decrease in the hydrodynamic size, increase in the zeta potential value with the addition of **TPE-V** molecules to CT-DNA, failure of TPE-V/cucurbit(8)uril complex to condense CT-DNA, and the enhanced DNA condensation ability of **TPE-V2** with two viologen units compared to **TPE-V1** with a single viologen unit confirm the importance of positively charged viologen units in the DNA condensation process. Initial cytotoxicity analysis on A549 cancer and WI-38 normal cells revealed that these DNA condensing agents are non-toxic in nature and hence could be utilized in further cellular delivery studies.

## Introduction

DNA condensation is the process of converting elongated, negatively charged double-helical random coils of DNA into smaller, charge-neutralized micro- or nano-sized compact structures with the help of condensing or compaction agents (Bloomfield, 1996; Bloomfield, 1997). The general strategies for inducing DNA condensation include developing attraction between individual DNA double strands by charge neutralization process or creating unfavorable interactions between DNA strands and the solvent (Gonzalez-Perez and Dias, 2009; Estévez-Torres and Baigl, 2011). Common DNA condensing agents include multivalent counterions of metals, polyamines, peptides, cationic polymers, dendrimers, surfactants, nanoparticles, carbon nanotubes, and small molecules (Gosule and Schellman, 1976; Arakawa et al., 2000; Matulis et al., 2002; Ganguli et al., 2004; Baigl and Yoshikawa, 2005; Liu et al., 2008; Huang et al., 2016; Fan et al., 2017; Cao et al., 2018; Gupta et al., 2019). Among the above-mentioned DNA condensing agents, cationic surfactants are the most commonly employed non-viral gene delivery vectors because of their highly cooperative binding behavior with DNA (Hayakawa et al., 1983; Dias et al., 2004; Marchetti et al., 2005). The condensation abilities of these cationic surfactants were found to increase with the increase in the size of hydrophobic nonpolar tails or the presence of a co-solute, promoting the surfactant aggregation (Diguet et al., 2010; Rudiuk et al., 2011). For example, Yaxun Fan and coworkers have demonstrated the calf-thymus DNA condensation induced by a star-shaped hexameric cationic surfactant, PAHB (Fan et al., 2017). With the addition of PAHB to CT-DNA, conformational changes in the DNA structure from the long coil to partially condensed cluster aggregates, globules on a string structure, and the subsequent formation of completely condensed globule-like structures were reported. Here, the electrostatic interaction between multiple positively charged PAHB and the negatively charged phosphate backbone of CT-DNA is the key factor that promotes the DNA condensation process.

Inspired by the concept of DNA condensation by cationic surfactants and using the design of its chemical structure, small cationic or amphiphilic organic molecules and their self-assembled supramolecular structures have also received significant attention because of their unique interactions with nucleic acids *via* groove binding, π-π stacking, and/or electrostatic interactions and subsequent condensation capabilities (Cassell et al., 1998; Liu et al., 2007; Yan et al., 2012; Minami et al., 2014; Nitta et al., 2015; Mondal et al., 2018). Functionalized fullerene derivatives are well explored and investigated for *in vitro* DNA condensation and *in vivo* gene delivery applications (Nakamura and Isobe, 2010; Minami et al., 2018). Recently, we have demonstrated a fullerene cluster assisted self-assembly of short DNA double strands into nanowires, DNA three-way junction structures into nanosheets and 2-dimensional nanonetworks, and the chiral organization of fullerene clusters on CT-DNA templates followed by concentration-dependent DNA condensation process (Vittala et al., 2017; Kulala Vittala and Joseph, 2018; Vittala et al., 2019). Here, we employed the unique hydrophilic-hydrophobic balance in the molecular structure of an aniline-functionalized fullerene derivative, F-An, in a 10% DMSO-PBS solvent system to prepare 3–5 nm fullerene nanoclusters, and their interactions with different DNA structures were demonstrated. In the case of CT-DNA, the highly fibrous network structure with ∼250 nm width breaks down to 50–100 nm small fibrous structures and subsequently to compact structures as a result of the F-An–DNA groove binding directed condensation process (Kulala Vittala and Joseph, 2018).

On the other hand, organic chromophores with unique photophysical and self-assembling properties are not well explored for DNA condensation studies because of their poor aqueous solubility and hence lesser biocompatibility for gene delivery applications. Recently, Yi Han et al. (2017) have reported a pyrene functionalized amphiphilic dendrimer with pDNA condensation properties accompanied by sensible fluorescence changes for monitoring the DNA binding process. Later, J. H. Mondal et al. (2018) have demonstrated the use of a water-soluble ethyl viologen-functionalized tetra-cationic perylenediimide derivative (PDEV) for an effective CT-DNA condensation process, using spectroscopic, morphological, and imaging techniques. Here, the fluorescence changes in the PDEV chromophore as a result of the conformational changes in the PDEV/CT-DNA complex were examined to study the DNA condensation process. As in this example, the development of promising DNA compaction agents based on organic chromophores with improved water solubility and significant changes in the absorption and fluorescence properties upon interaction with DNA can be utilized for absorption/fluorescence-based monitoring of the DNA condensation process. Herein, we report the design, synthesis, CT-DNA interaction, and condensation properties of two novel viologen-functionalized tetraphenylethylene (**TPE-V**) molecules. TPE chromophores are well known for their aggregation-induced emission (AIE) property and there are few reports on TPE-DNA conjugates for sensing and catalytic applications (Lou et al., 2014; Zhang et al., 2015; Geng et al., 2017; Krishnan et al., 2018; Rothenbühler et al., 2020). Here, we describe the interactions of random aggregates of these molecules in Tris buffer with CT-DNA through electrostatic/groove binding modes, which form an initial “beads-on-a-string” type assembly and subsequent completely charge-neutralized compact DNA structures at higher concentrations of TPE derivatives. The process of DNA condensation was examined using photophysical and morphological analysis techniques. Furthermore, the cytotoxicity analysis on A549 cancer and WI-38 normal cells revealed the non-toxic nature of these DNA condensing agents.

## Materials and Methods

All the materials, methods, synthetic procedures, characterization data, and supporting figures are included in the Supplementary Material.

## Results and Discussion

**SCHEME 1:**
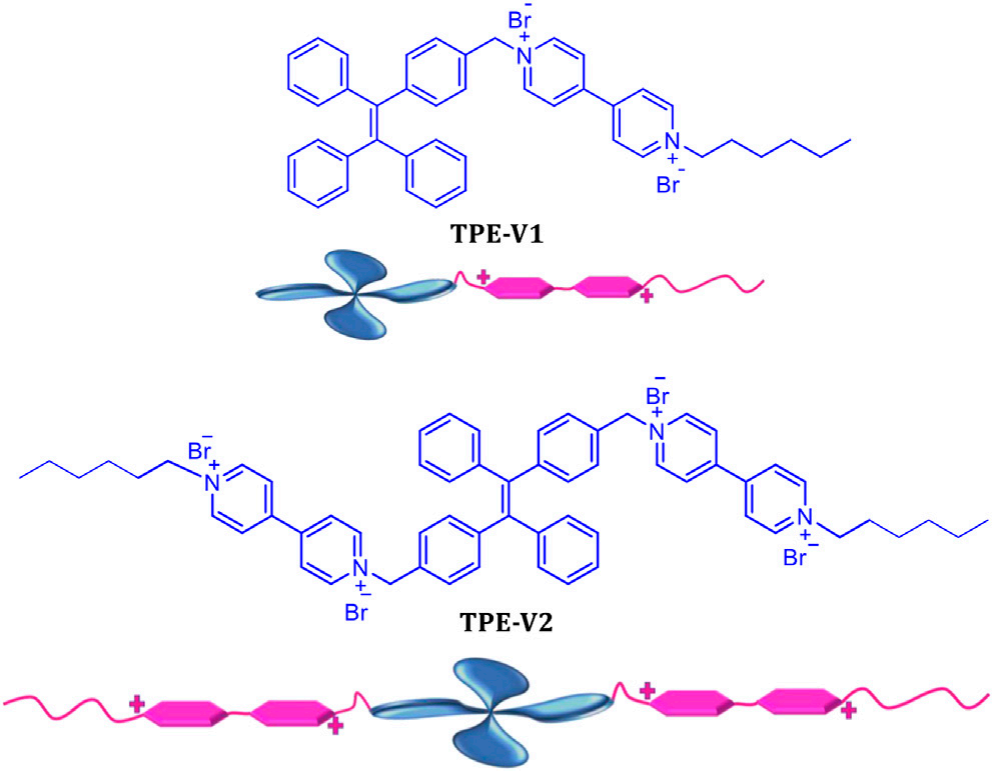
Chemical structure and pictorial representation of **TPE-V1** and **TPE-V2**.

To study the DNA interactions and induced DNA condensation properties with tetraphenylethylene chromophores, we synthesized two viologen-appended TPE derivatives, **TPE-V1** and **TPE-**V2 (Scheme 1). **TPE-V1** has one viologen group separated by a methylene spacer, whereas **TPE-V2** has two viologen units separated by a single methylene spacer from the TPE chromophore. The synthesis of **TPE-V1** and **TPE-V2** was achieved by a modified four-step synthetic procedure (Supplementary Schemes S1, S2), starting from McMurry coupling reaction between appropriately substituted benzophenone derivatives, and the final products were obtained in 79 and 83% yield, respectively (Liang et al., 2014; Ma et al., 2018).

The viologen moieties with the quaternary amine groups impart moderate aqueous solubility to the hydrophobic TPE core and are responsible for the strong DNA interactions *via* groove binding and electrostatic interactions (Joseph et al., 2003; Hvastkovs and Buttry, 2006; Zhang et al., 2011). Before studying DNA interactions, we have investigated the photophysical properties of **TPE-V1** and **TPE-V2** in Tris buffer (10 mM Tris buffer with 2 mM NaCl). TPE molecules possess unique aggregation properties in solvent mixtures characterized by significant changes in their photophysical properties. Methanol solutions of **TPE-V1** and **TPE-V2** exhibited absorption maxima around 315 nm with emission peaks corresponding to monomeric species (blue spectra in Supplementary Figures S1A,B). In contrast, in Tris buffer, a hypochromic shift was observed in the 315 nm absorption maxima along with the formation of 400 nm emission band, indicating the aggregation behavior (red spectra in Supplementary Figures S1A,B.

**FIGURE 1:**
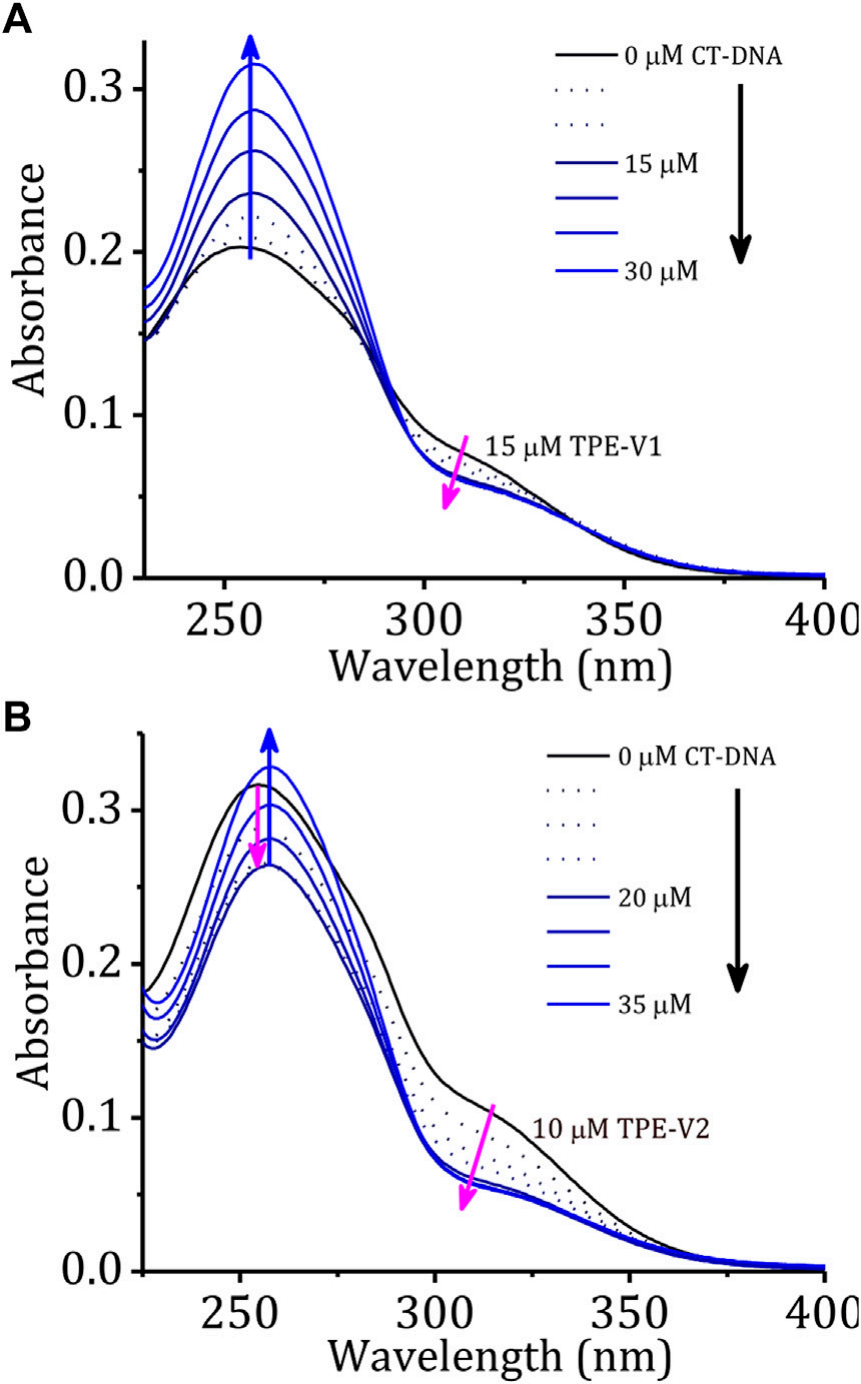
Changes in the absorption spectra of **(A) TPE-V1** (15 μM) and **(B) TPE-V2** (10 μM) in Tris buffer with the sequential addition of CT-DNA (every single addition makes 5 μM in the final volume).

Initially, the DNA interaction and consequent DNA condensation abilities of these aggregates with long double-stranded genomic CT-DNA were studied using UV-visible absorption and fluorescence emission spectroscopy. Upon sequential addition of CT-DNA to **TPE-V1** in Tris buffer, the characteristic absorption band at 315 nm showed a consistent hypochromic shift up to one equivalent addition of CT-DNA (Figure 1A, CT-DNA concentration is per single nucleotide with a unit negative charge), suggesting further aggregation of **TPE-V1/CT-DNA** complex. Similarly, sequential addition of CT-DNA to **TPE-V2** in Tris buffer showed the aggregation of **TPE-V2/CT-DNA** complex (Figure 1B) with a consistent hypochromicity in the 260 and 315 nm absorption bands up to a 1:2 equivalent ratio of **TPE-V2** to CT-DNA. Upon further increasing the concentration of CT-DNA, a negligible decrease in the 315 nm absorption band and considerable enhancement in the 260 nm absorption band were observed. The interactions evident up to 1:1 ratio for **TPE-V1** and 1:2 ratio for **TPE-V2** imply a quantitatively higher amount of CT-DNA interacting with **TPE-V2** than that with **TPE-V1**. These results suggest the charge neutralization process accomplished by the interaction of positively charged viologen head group in **TPE-V** molecules with the negatively charged phosphate backbone of CT-DNA leading to aggregation of **TPE-V/CT-DNA** complex, as indicated by the hypochromicity of the 315 nm absorbance band.

A comparison of changes in the absorption spectra of **TPE-V** molecules and a blank solution (buffer alone) at 260 nm with the sequential addition of CT-DNA (Supplementary Figure S2) showed a steady enhancement at 260 nm for the blank solution due to the increased absorption of the DNA. On the other hand, due to the interactions with the **TPE-V** molecules and subsequent aggregation, the absorption around 260 nm showed an initial weak enhancement up to 1:1 ratio for **TPE-V1** and a decrease up to 1:2 ratio for **TPE-V2**. At higher concentrations of CT-DNA, a gradual enhancement due to the increase in free CT-DNA was observed. In agreement with absorption changes, the corresponding fluorescence profiles also showed a slight enhancement with the sequential addition of CT-DNA up to an equivalent ratio of 1:1 for **TPE-V1** and 1:2 for **TPE-V2** (Supplementary Figures S3A,B). These differences could be attributed to the initial electrostatic interaction and subsequent charge neutralization leading to aggregation of **TPE-V** along with the CT-DNA template, which eventually will lead to condensed structures.

**FIGURE 2:**
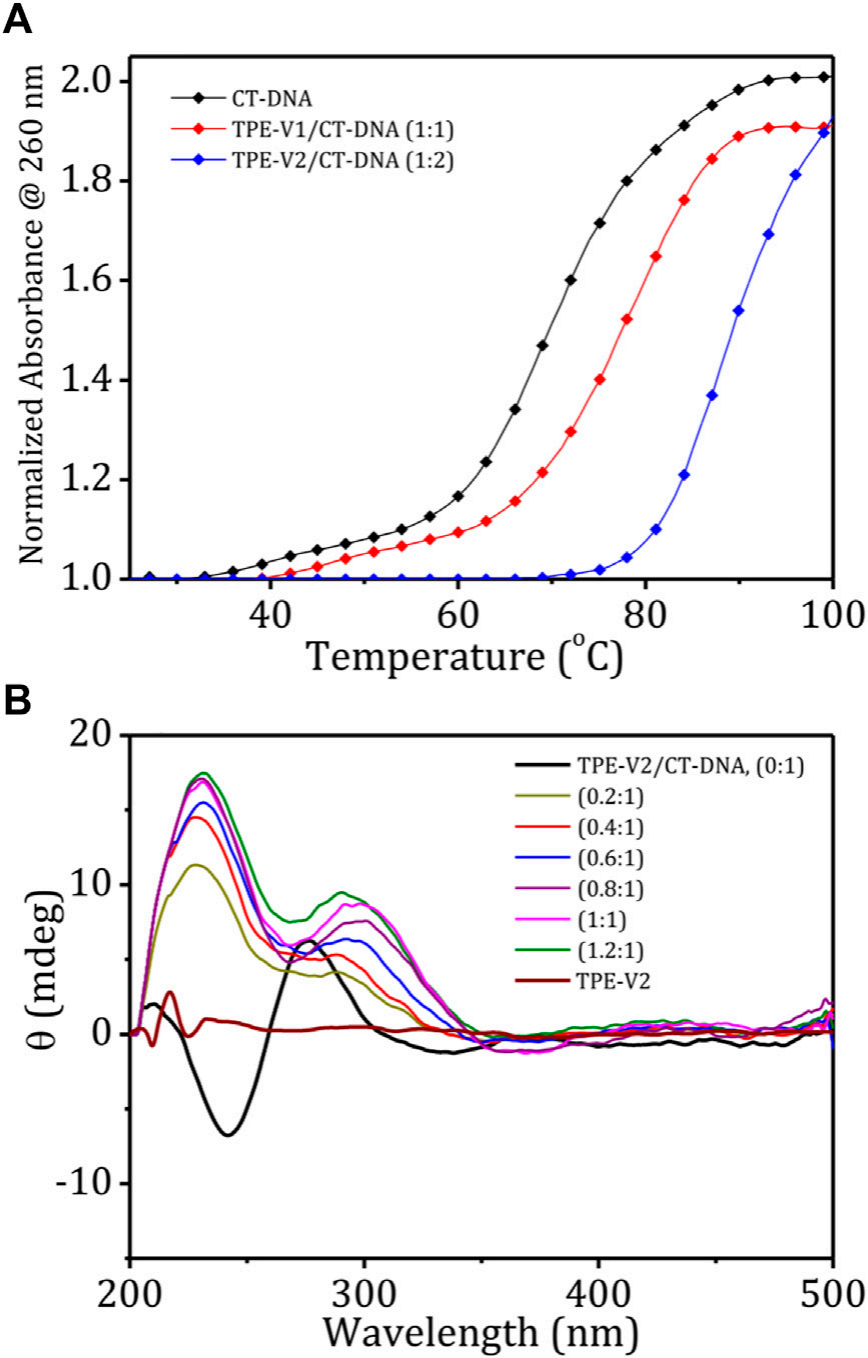
**(A)** Thermal denaturation curves of CT-DNA in the absence and presence of **TPE-V1** and **TPE-V2**; **(B)** CD spectra of CT-DNA (100 μM), **TPE-V2** (100 μM) and changes in the CD spectrum of CT-DNA with the sequential addition of **TPE-V2** (0–1.2 M equivalent).

The stability and structural changes in the duplex CT-DNA upon interaction with **TPE-V** molecules were analyzed by performing thermal denaturation and circular dichroism studies. Figure 2A shows the melting curves constructed by recording the changes in absorption at 260 nm with an increase in the temperature from 20 to 100°C and a heating rate of 1°C per minute. The melting temperature of CT-DNA increases from 68 to 78°C in the presence of **TPE-V1** (1:1 complex) and to 88°C in the presence of **TPE-V2** (1:2 complex). The enhanced thermal stability of **TPE-V/CT-DNA** complexes indicates that the **TPE-V** molecules interact efficiently with CT-DNA but with differential strengths. The higher thermal stability of **TPE-V2/CT-DNA** compared to **TPE-V1/CT-DNA** could be attributed to the presence of two viologen units in the former, giving rise to a quantitative enhancement in DNA binding. These results suggest the cationic **TPE-V** molecule–directed DNA compaction results in condensed structures with enhanced thermal stability for CT-DNA (Mondal et al., 2018).

Further, the structural changes in CT-DNA were examined using CD spectroscopy by reverse titrating **TPE-V** molecules against a solution of CT-DNA (Supplementary Figure S4; Figure 2B). The CD spectrum of CT-DNA displayed a typical B-form duplex DNA signature with a characteristic 247 nm negative band corresponding to polynucleotide helicity and 277 nm positive band originating from base stacking. In contrast, CD spectra of CT-DNA in the presence of increasing concentrations of **TPE-V** molecules (0–1.2 M equivalent) showed a gradual decrease in the 247 nm band and the formation of a slightly red-shifted and broadened band at 305 nm from the initial 277 nm band, extending up to 350 nm. This considerable change observed in the characteristic CD signal of B-form CT-DNA to a signal above 300 nm indicates the condensation of DNA into particles that can scatter light (Kypr et al., 2009). In addition, the newly formed induced circular dichroism (ICD) band in the region of the absorption of **TPE-V** molecules also suggests the chiral organization of **TPE-V** molecules on the CT-DNA template. Compared to the **TPE-V1** (Supplementary Figure S4), the ICD band formation was more evident with the sequential addition of **TPE-V2** to CT-DNA (Figure 2B), which could be attributed to the enhanced interaction of **TPE-V2** with CT-DNA.

**FIGURE 3:**
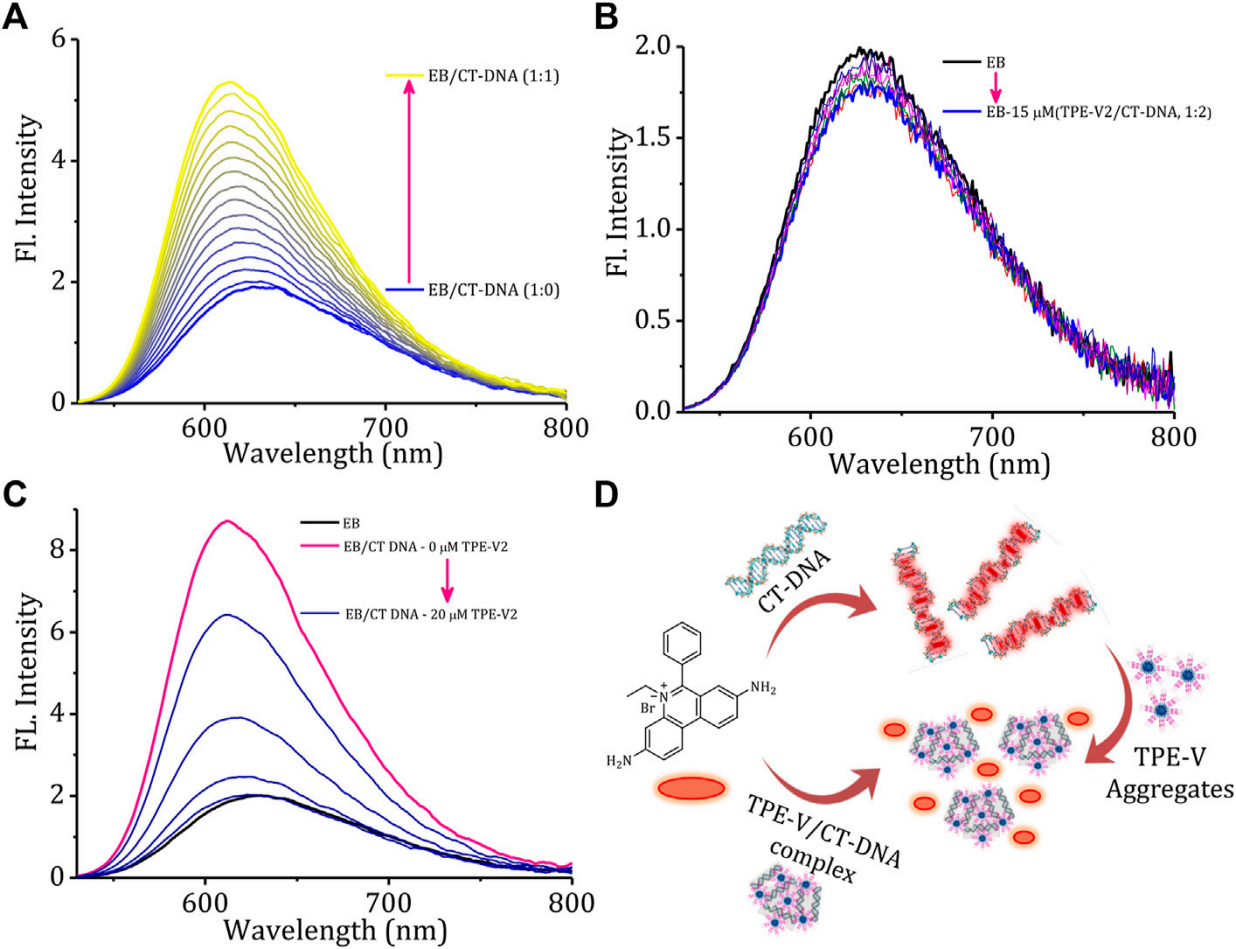
Changes in the emission spectra of EB (15 μM) with increasing concentrations of **(A)** CT-DNA (0–15 μM) and **(B) TPE-V2/CT-DNA** (1:2 complex, 0–15 μM). **(C)** Changes in the emission spectra of EB/CT-DNA complex (1:2) with the sequential addition of **TPE-V2** (0–20 μM) and **(D)** schematic representation of the overall EB intercalation and displacement studies.

Fluorescence displacement assay using ethidium bromide (EB) as the DNA probe is a suitable method for identifying the mode of binding between small molecules and DNA. EB shows enhancement in the fluorescence intensity upon intercalation between the base pairs of duplex DNA structure, thereby providing structural information about DNA. To understand the structural changes and mode of binding in **TPE-V/CT-DNA** complexes, initially, we carried out a typical titration experiment of CT-DNA against EB, which shows the enhancement in fluorescence intensity upon intercalation of EB into CT-DNA (Figure 3A). A similar titration experiment performed with **TPE-V1/CT-DNA** complex and **TPE-V2/CT-DNA** complex against EB has shown only an initial marginal decrease in the fluorescence emission spectra (Supplementary Figure S5A; Figure 3B). Therefore, the failure of EB to intercalate between the base pairs of CT-DNA in the **TPE-V/CT-DNA** complexes suggests conformational changes in long double-helical DNA structure upon interaction with **TPE-V** molecules.

In addition, several examples of the interaction of cationic ligands with EB/DNA complexes resulting in DNA condensation and subsequent displacement of EB from the DNA complex with quenching of EB fluorescence were also reported in the literature (Cain et al., 1978; Szumilak et al., 2016). TPE-V molecules were titrated against a preincubated solution of the EB/CT-DNA complex to verify the DNA condensation process. As observed in the above experiment (Figure 3A), the fluorescent intensity of EB increases upon incubation with CT-DNA and shows a 4-fold enhancement at a 1:2 equivalent ratio. Supplementary Figure S5B and Figure 3C show the changes in the emission spectra of EB upon titrating **TPE-V** against EB/CT-DNA (1:2) complex. The addition of **TPE-V1/TPE-V2** to EB/CT-DNA complex showed gradual quenching of the EB fluorescence, suggesting the displacement of intercalated EB from CT-DNA due to DNA conformational changes upon interaction with **TPE-V** molecules. These results of ethidium bromide displacement assays (schematic representation, Figure 3D) strongly support the possible condensation of the double-helical DNA structure of CT-DNA upon interaction with positively charged **TPE-V** molecules. Though quenching in fluorescence intensity was observed in the case of both **TPE-V** molecules, the extent of quenching was greater for **TPE-V2** (20 μM) than for **TPE-V1** (30 μM), thereby providing a measure of the DNA condensation ability of these **TPE-V** molecules.

To gain further insight into the interacting units in **TPE-V** molecules with CT-DNA, DNA binding experiments were carried out in the presence of cucurbit(8)uril [CB[8]]. CB[8] is a well-known host for viologen guests, with the ability to completely encapsulate the guest inside its cavity (Lee et al., 2003; Zhang et al., 2016). For this experiment, we titrated CT-DNA against the preformed CB[8]/**TPE-V** host-guest complex. The UV-visible absorption spectra of CB[8]/**TPE-V1** (1:1) complex showed slightly lower absorbance values at 255 and 315 nm than those of **TPE-V1** alone (Supplementary Figure S6A) and also displayed an enhanced fluorescence emission (Supplementary Figure S6B). Sequential addition of CT-DNA to this complex showed negligible changes in the UV-visible absorption and fluorescence emission, as expected. Similarly, the absorption and fluorescence spectra of CB[8]/**TPE-V2** (2:1) complex also were not affected by sequential addition of CT-DNA, except for the initial decrease in absorption and enhancement in fluorescence emission spectra compared to the **TPE-V2** alone (Supplementary Figures S6C,D). These results imply that the strong complexation with CB[8] results in complete masking of viologen units in the CB[8]/**TPE-V** complex and prevents the binding of viologen moiety with CT-DNA. Hence, the CT-DNA interaction and subsequent condensation observed with **TPE-V** molecules are primarily due to the presence of positively charged viologen groups and their interaction with CT-DNA through electrostatic and groove binding modes.

**FIGURE 4:**
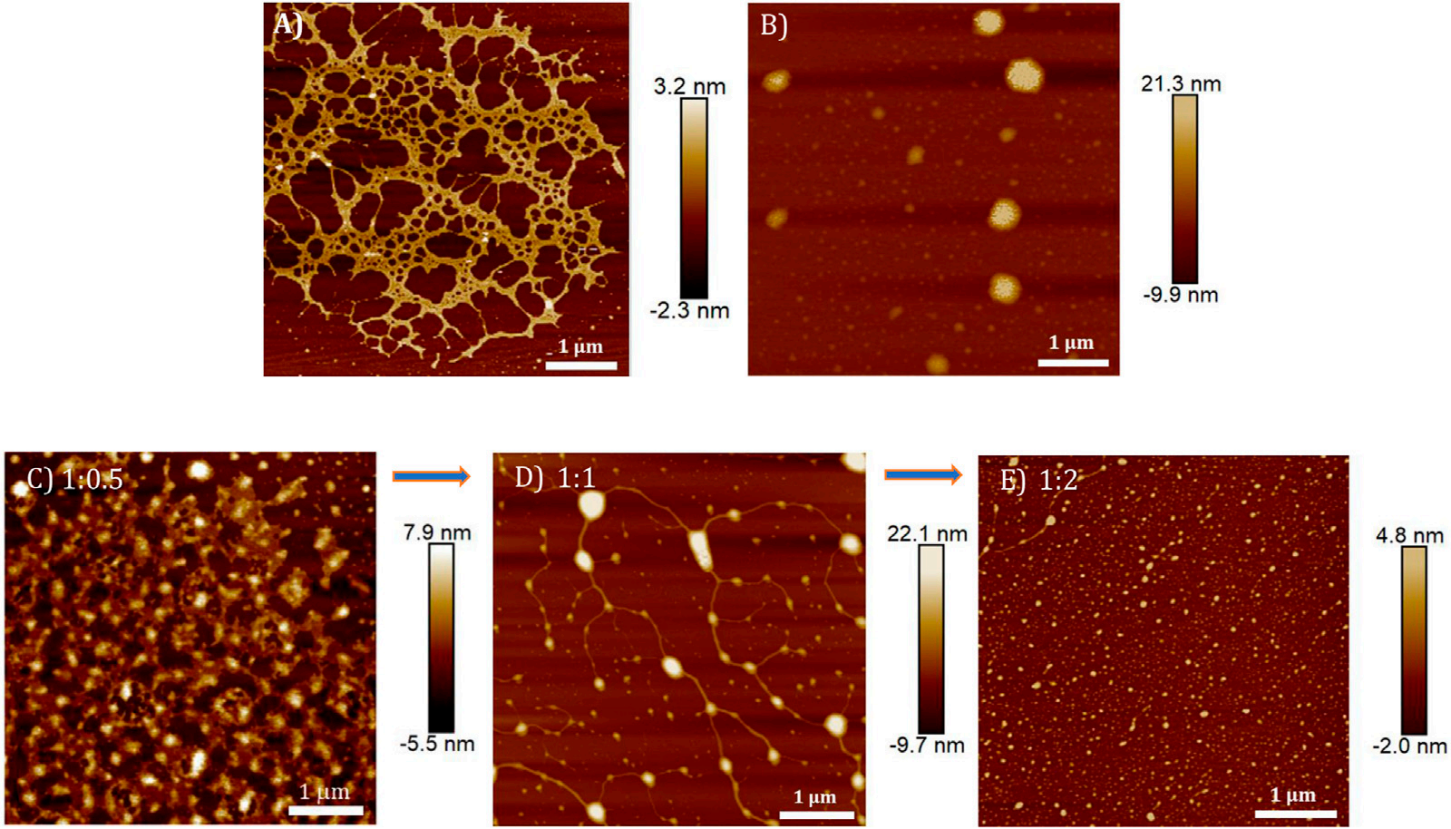
AFM images of **(A)** CT-DNA (15 μM), **(B) TPE-V1** (15 μM) and **TPE-V1/CT-DNA** complex at different CT-DNA to **TPE-V1** ratios, **(C)** 1:0.5, **(D)** 1:1 and **(E)** 1:2.

The DNA condensation process was further investigated in detail by monitoring the morphology changes associated with the **TPE-V/CT-DNA** interaction using AFM, TEM, and DLS techniques. Sample concentrations in the micromolar range used for the UV-visible absorption studies were chosen for AFM and TEM analysis, and the ratios represent CT-DNA to **TPE-V** molar ratios. In contrast, higher concentrations used for CD studies were chosen for DLS analysis. CT-DNA alone displayed fibrous network-like structures with 200–300 nm width (Figure 4A), while **TPE-V1** alone displayed random, spherical aggregates with 350 nm diameter for the largest aggregate (Figure 4B). On the other hand, **TPE-V1/CT-DNA** complex displayed a hybrid morphology of **TPE-V1** and CT-DNA, exhibiting an entangled network structure (Figure 4C) at a 1:0.5 ratio and “beads-on-a-string” arrangement (Figure 4D) similar to the structure of chromatin fibers found in the chromosomes of eukaryotic cells at a 1:1 ratio. In the “beads-on-a-string” arrangement, the strings (fibers) showed an average width of 80 nm, while the beads (spherical aggregates on fibers) had an average width of 110 nm. Further, at higher concentrations of **TPE-V1** in **TPE-V1/CT-DNA** complex (1:2), the AFM images showed spherical aggregates with an average diameter of 47 nm (Figure 4E and Supplementary Figure S7A), indicating complete condensation of the complex.

**FIGURE 5:**
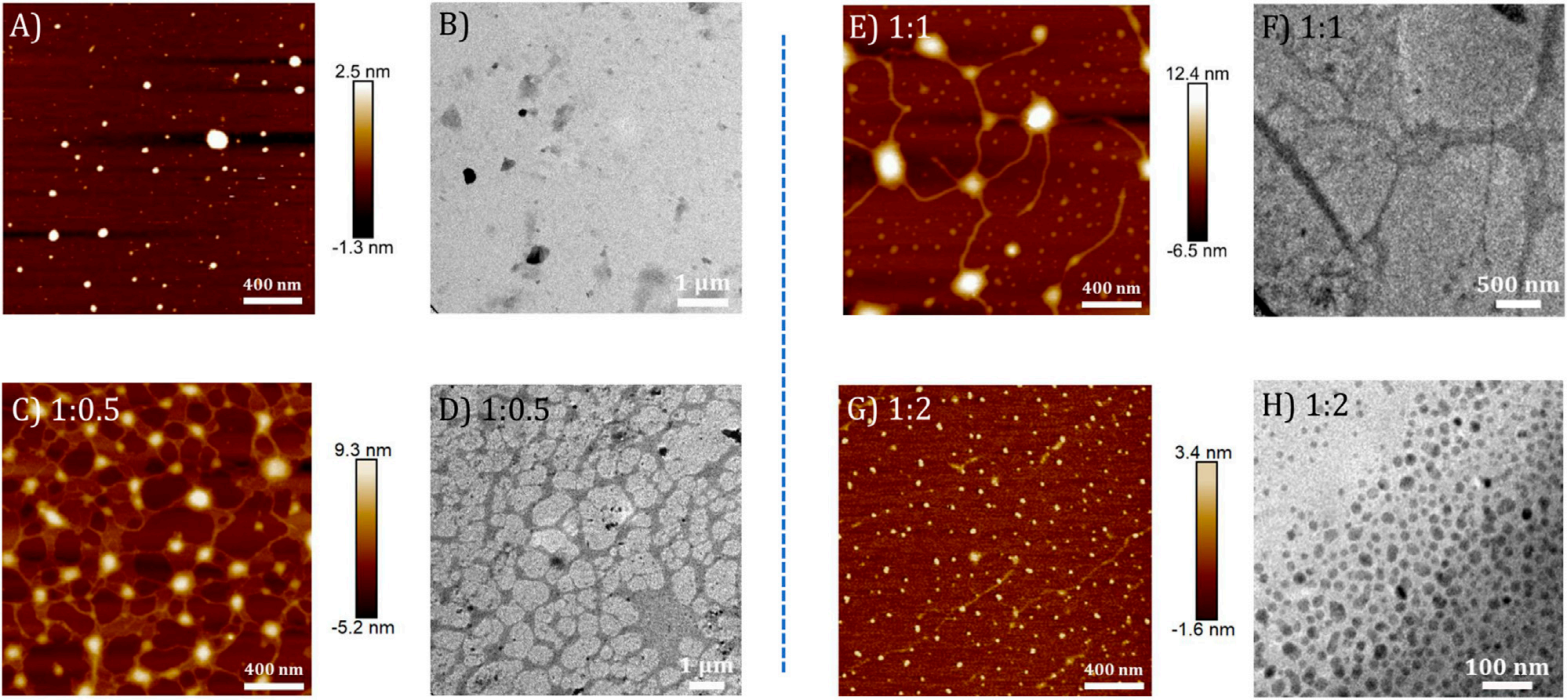
AFM and corresponding TEM images of **(A,B) TPE-V2** (10 μM) and **TPE-V2/CT-DNA** complex at different CT-DNA to **TPE-V2** ratios: **(C,D)** 1:0.5, **(E,F)** 1:1, and **(G,H)** 1:2.

Similarly, the morphologies of **TPE-V2/CT-DNA** complex at different **TPE-V2** to CT-DNA ratios displayed initial formation of intertwined network structures at a 1:0.5 ratio, while beads-on-a-string (1:1) and condensed spherical structures (1:2) are formed at higher **TPE-V2** concentrations. In detail, the AFM morphologies at 1:0.5 ratios of **TPE-V2/CT-DNA** complex showed the disruption of the fibrous network structures of CT-DNA (Figure 4A) to intertwined network structures (Figure 5C) with morphological features of both CT-DNA and **TPE-V2** (Figure 5A). Upon further increasing the concentration of **TPE-V2** in **TPE-V2/CT-DNA** complex, enhanced disruption of CT-DNA fibrous structures by **TPE-V2** leading to a “beads-on-a-string” type of arrangement (Figure 5E) at a 1:1 ratio and completely condensed spherical **TPE-V2/CT-DNA** structures with an average size of 43 nm at a 1:2 ratio (Figure 5G and Supplementary Figure S7B) was observed in the AFM images. In agreement with the AFM results, TEM images of CT-DNA (Supplementary Figure S7C) and **TPE-V2** (Figure 5B) displayed networked fibrous structures and random aggregated structures, respectively, as observed in AFM analysis. Similarly, in agreement with AFM morphologies, the **TPE-V2/CT-DNA** complex with increasing concentrations of **TPE-V2** showed the structural transition from the networked fibrous CT-DNA structure to condensed spherical structures of **TPE-V2/CT-DNA** complex with 30–40 nm in size (Figures 5D,F,H). In addition, a comparison of the morphological features of the **TPE-V2/CT-DNA** complex and the **TPE-V1/CT-DNA** complex at a 1:0.5 ratio indicates that the progression to “beads-on-a-string” type arrangement was more pronounced in the case of **TPE-V2**, which further supports the enhanced DNA interaction ability of **TPE-V2**. The observed AFM and TEM results reveal, at lower concentrations of **TPE-V** in CT-DNA, the electrostatic interactions between positively charged viologen moiety of aggregated **TPE-V** and fibrous network of negatively charged CT-DNA structures lead to the separation of fibrous DNA networks and disruption of larger **TPE-V1** aggregates to smaller ones. Under these conditions, the electrostatically bound **TPE-V** aggregates tend to form bead-type structures on the long CT-DNA templates, giving rise to the ICD for **TPE-V**. In contrast, at a higher concentration of **TPE-V**, complete condensation of CT-DNA is observed due to effective charge neutralization. Thus, upon increasing the concentration of **TPE-V** in the **TPE-V/CT-DNA** complex to 1:2, the interaction possibilities between DNA and molecule increase, leading to the disruption of larger aggregates of **TPE-V** along with effective charge neutralization of the negatively charged DNA backbone. Consequently, the “beads-on-a-string” structures with DNA chains and **TPE-V** aggregates completely condense into spherical structures of 40–50 nm in size. Furthermore, EDAX analysis of **TPE-V2/CT-DNA** complex at a 1:2 ratio of CT-DNA to **TPE-V2** spots the occurrence of P atoms (Supplementary Figure S7D), confirming the presence of DNA in the condensed spherical structures observed in TEM.

**FIGURE 6:**
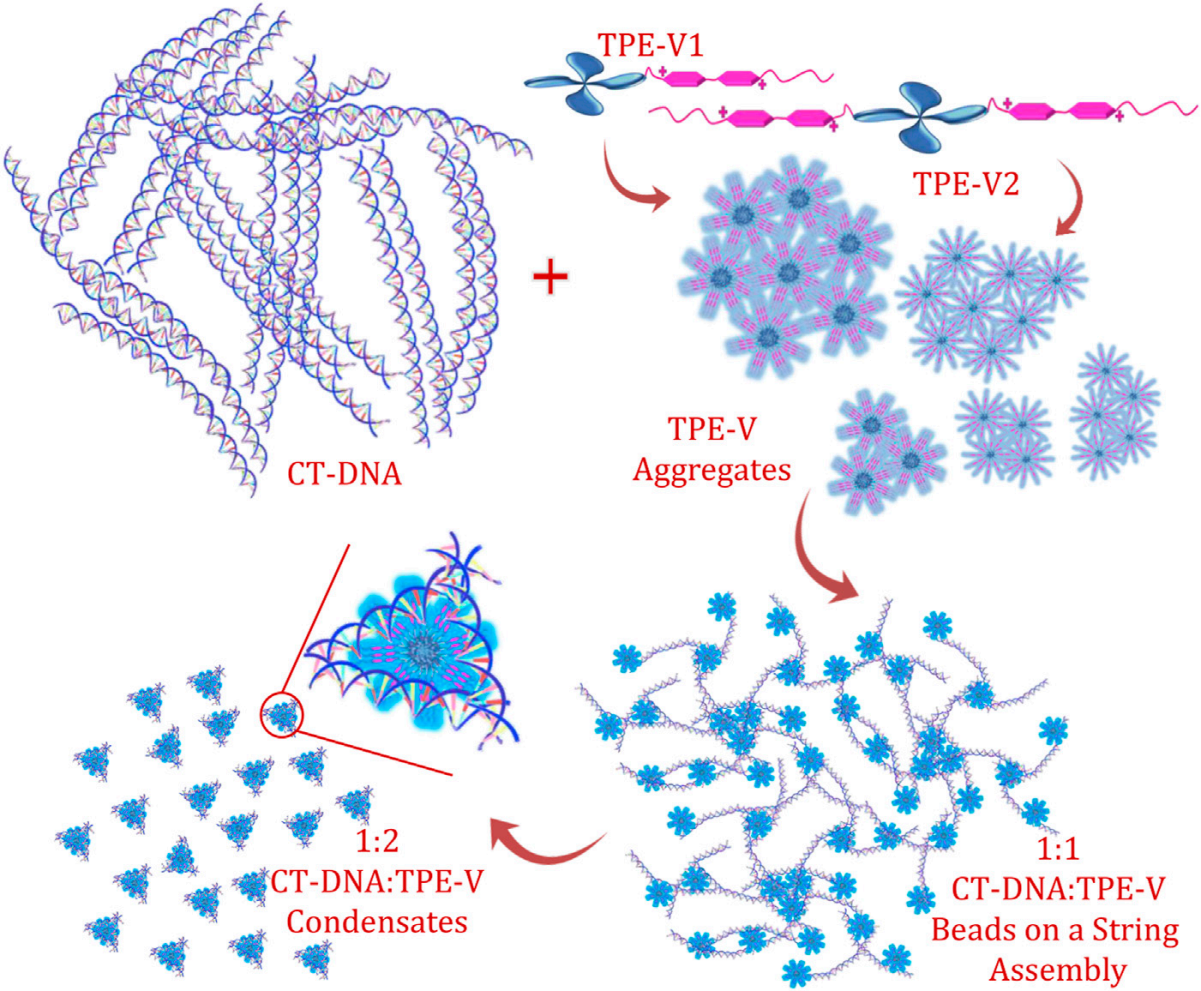
Schematic representation of the CT-DNA interaction and condensation process by **TPE-V** molecules.

Among the two **TPE-V** molecules, **TPE-V2** with two viologen units interacts with CT-DNA even at lower concentrations (1:0.5) compared to **TPE-V1** (1:1), which further leads to the synergistic disruption of both CT-DNA and **TPE-V** assemblies into **TPE-V/CT-DNA** condensates. Figure 6 shows a schematic representation of the proposed mechanism of the DNA condensation process by **TPE-V** molecules. To gain further insight into the DNA condensation process, DLS measurements were carried out to understand the zeta potential and size changes accompanying the process (Supplementary Figures S8A,B). CT-DNA alone in Tris buffer showed −35 mV to −40 mV zeta potential and 700–750 nm hydrodynamic radius. With the sequential addition of **TPE-V** molecules, the zeta potential values showed a gradual increase toward positive values and hydrodynamic size showed a steady decrease to 100–200 nm. Since CT-DNA is a highly networked structure, the hydrodynamic radius we observe here only gives a rough estimate of size in solution. However, it still can indicate the changes upon **TPE-V/CT-DNA** interaction leading to spherical condensates. These opposite trends in zeta potential and size further support the charge neutralization directed DNA condensation resulting in **TPE-V/CT-DNA** condensates.

**FIGURE 7:**
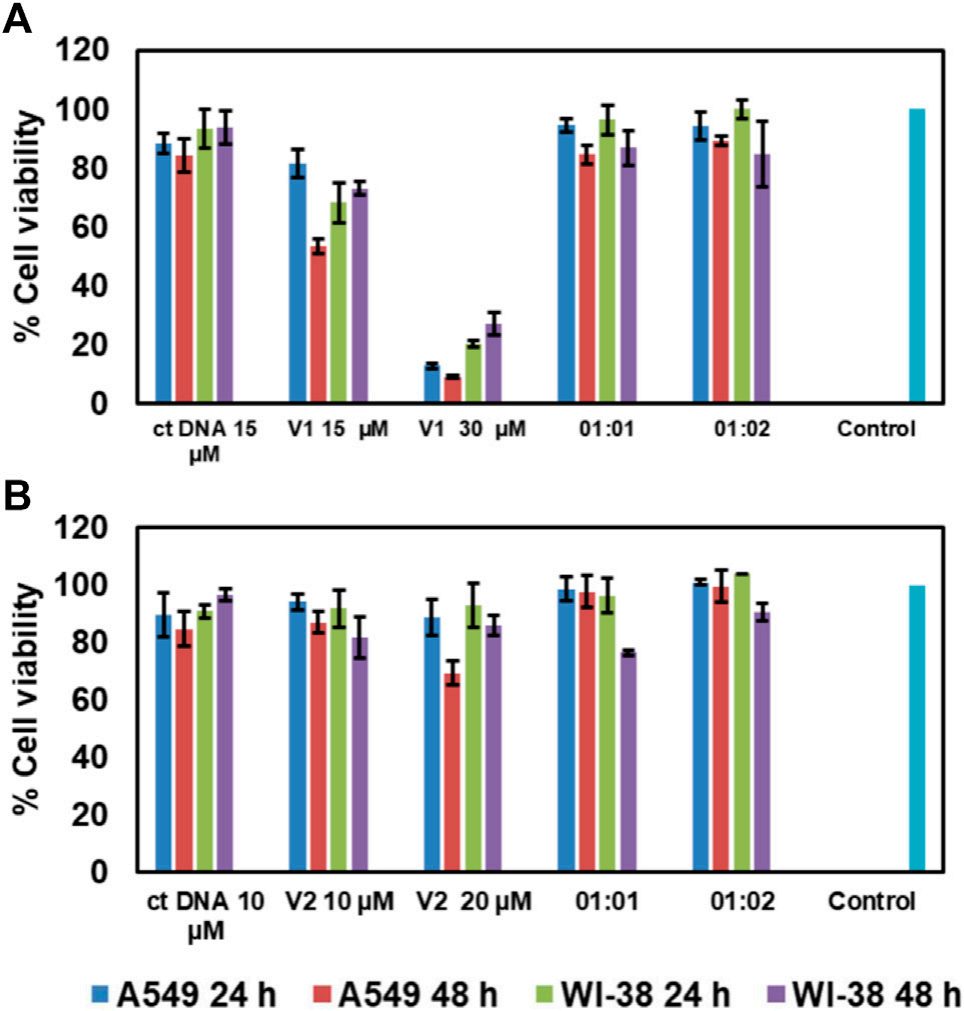
Cell viability assay of A549 and WI-38 cells in the presence of **(A)** **TPE-V1**/CT-DNA and **(B)** **TPE-V2**/CT-DNA assemblies and their control experiments after 24 and 48 h incubation.

Cytotoxicity analysis is an important initial study to check the biological *in vitro* applicability of drugs and chemicals, giving information about the toxicity of these chemicals on cells. A cell viability value greater than 90% indicates their biocompatibility and potential in intracellular drug and gene delivery. To check the cytotoxicity of our **TPE-V** DNA condensing agents, an MTT assay was conducted. The cytotoxicity of **TPE-V**/**CT-DNA** condensates was studied with A549 and WI-38, lung adenocarcinoma, and normal lung fibroblast cells, respectively. Figures 7A,B show the cell viabilities of CT-DNA, **TPE-V** aggregates, and **TPE-V**/**CT-DNA** complexes at two different ratios (1:1 and 1:2 of CT-DNA to **TPE-V**) after 24 and 48 h incubation. The concentrations of CT-DNA and **TPE-V** derivatives are the same as those used for absorption, emission, and morphological studies. At these concentrations, CT-DNA displayed less cytotoxicity with more than 80% cell viability in both cell lines at 24 and 48 h. In the case of **TPE-V1**, slight toxicity was observed for 15 µM samples in A549 cell line at 48 h and 30 µM showed significant toxicity with less than 30% viability toward both cells lines at 24 and 48 h. In contrast, **TPE-V2** was not much toxic to both cell lines at the said time points, showing more than 70% viability. Interestingly, **TPE-V/CT-DNA** complexes displayed enhanced cell viability of 80–90% or greater, showing their applicability *in vitro* models. These results suggest that the “beads-on-a-string” type assembly (1:1) and spherical condensates (1:2) of **TPE-V/CT-DNA** have superior cell viabilities compared to their precursors, and hence, these **TPE-V** molecules are reasonably good DNA condensing agents for non-viral gene delivery applications.

## Conclusion

In summary, we have reported the synthesis of two viologen-functionalized tetraphenylethylene (**TPE-V**) molecules, which can replace the common DNA condensing agents, such as counterions, cationic polymers, and polyamines, with an additional property of absorbance/fluorescence-based tracking of the DNA condensation and gene delivery process. Moreover, we have demonstrated the CT-DNA binding, subsequent DNA condensation, and cytotoxicity studies of these **TPE-V** molecules using optical, morphological, and MTT assay techniques. The enhanced stability observed in the thermal denaturation studies and ICD band observed in the CD studies of CT-DNA in the presence of **TPE-V** derivatives suggested the DNA condensation process. Further, the corresponding morphological analysis revealed the synergistic dis-assembly of the random aggregates of **TPE-V** molecules and the fibrous CT-DNA structure in to an initial, partially condensed “beads-on-a-string” type assembly at lower concentrations of **TPE-V**, and completely condensed compact DNA nanoparticles of 40–50 nm in size at higher concentrations of **TPE-V**. The role of positively charged viologen units in directing the DNA condensation process was verified by zeta potential measurements and CB[8] encapsulation studies, which further confirmed that the electrostatic interaction and groove binding induced effective DNA condensation process by **TPE-V** molecules. The *in vitro* biological applicability of **TPE-V/CT-DNA** condensates tested by performing MTT assay on A549 cancer and WI-38 normal cells revealed the non-toxic nature of **TPE-V1** and **TPE-V2** CT-DNA condensates. In brief, this report highlights the development of small fluorescent organic chromophores as non-viral gene delivery vectors, which can bind with DNA through non-covalent interactions, such as groove binding and electrostatic binding, to achieve a controlled DNA condensation process with practical cell viability.

## Data Availability

The original contributions presented in the study are included in the article/Supplementary Material; further inquiries can be directed to the corresponding author.
