# Supplementary material for: DNA Condensation Triggered by the Synergistic Self-Assembly of Tetraphenylethylene-Viologen Aggregates and CT-DNA
Source: Front Chem. 2021 Jul 22;9:716771. doi: 10.3389/fchem.2021.716771 (PMC8341308; doi:10.3389/fchem.2021.716771)
Supplement: Supplementary file 1 [file DataSheet1.pdf]

frontiers

Supplementary Material

DNA Condensation Triggered by the Synergistic Self-Assembly of Tetraphenylethylene-Viologen Aggregates and CT-DNA

Sajena Kanangat Saraswathi[1,2], Varsha Karunakaran[1,2], Kaustabh Kumar Maiti[1,2] and Joshy Joseph[1,2*]

[1]*Photosciences and Photonics Section, CSIR-National Institute for Interdisciplinary Science and Technology, Thiruvananthapuram 695 019, India*

[2]*Academy of Scientific and Innovative Research (AcSIR), Ghaziabad 201002, India*

[*]**Correspondence:** joshy@niist.res.in

1. Materials and materials

All chemicals and solvents used for organic synthesis were purchased from Sigma Aldrich, Spectrochem and TCI, and were used without further purification. DMEM medium, Fetal Bovine Serum, Trypsin and Antibiotics used for cell culture were purchased from HiMedia and, MTT and HBSS used in cell viability assay were purchased from Sigma Aldrich. A549 (human lung adenocarcinoma) was procured from American Type Culture Collection (ATCC), Manassas, U.S. and WI-38 (Human normal lung fibroblast) was obtained from CSIR-IICB, Kolkata, India. TLC analysis were performed with Merck silica gel plates on aluminium sheets and column chromatography were performed using 230-400 mesh silica gel. ^{1}H NMR (500 MHz) and ^{13}C NMR (125 MHz) were performed in deuterated chloroform, methanol and DMSO with tetramethylsilane (TMS) (δ = 0 ppm for 1H and δ = 77 ppm for 13C) as an internal standard using Bruker Avance DPX spectrometer. Deionized Milli Q water (18.2 MΩ.cm) was used for all the studies.

The UV-visible absorption and fluorescence measurements were performed on Shimadzu UV-2600 spectrophotometer and SPEX FLUOROLOG-3 (FL3-221) spectrofluorimeter equipped with a 450 W Xenon arc lamp, respectively. All the optical measurements were carried out in 1 cm quartz cuvette at 25 ± 1 ^{o}C. UV-visible absorption and fluorescence emission studies of DNA binding experiment were carried out by the sequential addition of CT-DNA samples in to **TPE-V** derivatives in tris buffer (total dilution factor due to added aliquots is ~1.04). After each sequential addition of CT-DNA, the samples were mixed well and incubated at room temperature for 2-3 minutes before measurements. Ethidium bromide intercalation/displacement assays were performed in two ways. Initially by the sequential addition of **TPE-V/CT-DNA** complexes into EB solutions to check the intercalation possibilities of EB into CT-DNA structures in the **TPE-V/CT-DNA** complexes. And later by the addition of **TPE-V** derivatives into a EB intercalated CT-DNA complex to verify the condensation abilities of **TPE-V** derivatives. Fluorescence emission spectra of EB experiments were recorded using an excitation wavelength of 515 nm.

CD studies were performed at room temperature using Jasco J-810 spectropolarimeter and DLS analysis of the hydrodynamic size and zeta potential measurements were performed using Zetasizer (Malvern Nano ZS) operating with a He-Ne laser at a wavelength of 633 nm. 100 mM samples of CT-DNA and corresponding concentration ratios of **TPE-V** derivatives were used for CD and DLS measurements. In DLS analysis, each measurement was performed in triplicate, with 10-15

measurements in each run and the mean value is used to construct the plot of size, zeta-potential versus concentration of **TPE-V**.

CT-DNA samples were prepared by dissolving the commercially available (Sigma Aldrich) white fibrous mass in de-ionized Milli Q water. Initially, the solution was vortexed for 2-3 minutes and allowed to stand overnight at 0-4 ^oC to completely dissolve the fibrous structure. Later, the samples were filtered through 0.45 mm syringe filters and concentrations were calculated using the average ε value (6600 M^{-1} cm^{-1}) of single nucleotide at 260 nm. All the DNA-binding studies described in this work are carried out in 10 mM Tris buffer containing 2mM NaCl. 1 mM stock solutions of TPE-V were prepared by adding Milli Q water to appropriately weighed samples and heated to dissolve. From the hot stock solutions, concentrations required for studies were syringed out into cuvette and diluted to 1mL with Tris buffer. These solutions of TPE-V were incubated at room temperature for 30-40 minutes before performing DNA binding studies. This incubation time were chosen by monitoring the absorption changes to get a constant absorption for the aggregate band of TPE-V.

AFM/TEM sample preparations

Samples for AFM analysis were prepared by drop-casting 5-7 μL solution over freshly cleaved mica surface, followed by slow air drying. The images were recorded at ambient conditions using a BRUKER MULTIMODE AFM operating in the tapping mode. NT-MDT-NSG series, TiN cantilever tips with 299 kHz resonance frequency was used for the analysis. TEM samples were prepared by drop-casing 20-30 μL samples over carbon coated copper grid (400 meshes, Ted Pella) and air dried for 2 days at ambient conditions followed by vacuum drying before imaging. TEM images were recorded with JEOL-JEM0310 microscope with an accelerating voltage of 100 kV and the samples were imaged using a Hamamatsu ORCA charge-coupled device (CCD) camera.

Cell culture and MTT assay

Cells were grown and maintained in Dulbecco's modified Eagle's medium (DMEM, Himedia) added with 10% fetal bovine serum (FBS) and 1% Antibiotics Antimycotic solution (1X) at 37 °C with 5% CO_2. The cells were trypsinised and seeded in a 96 well flat bottom microlitre plate at a density of 10^4 cells per well in 100 μL complete DMEM complete medium. The cells were incubated at 37 °C with 5% CO_2 for 24 hrs enabling the cells to adhere. Later, cells were treated with 100 μL samples of CT-DNA (10 μM and 15 μM), TPE-V1 (15 μM and 30 μM), TPE-V2 (10 μM and 20 μM), 1:1 and 1:2 CT DNA condensates of TPE-V (diluted in serum free DMEM medium) followed by incubation for 24 h and 48 h. Afterwards, these cells were treated with 100 μL MTT (3-(4,5-dimethylthiazol-2-yl)-

2,5-diphenyl tetrazolium bromide) solution in HBSS (Hanks' Balanced Salt solution, 0.5 mg/ml). After 3 hrs, the solution in the wells were removed and 100 µL DMSO was added to dissolve the formazan crystals formed in the wells. The crystals were completely dissolved by giving a gentle rocking to the culture plates for 20 min and using a microplate reader (BioTek, Synergy H1M), the optical density at 570 nm was measured. Using these optical density values, obtained from different wells, the relative cell viability was calculated (Absorbance of [treated/control] x 100).

2. Synthesis and characterization

2.1. Synthesis of 1-hexyl-([4, 4'-bipyridine]-1-ium)bromide, (1) (Causin and Saielli, 2009)

To a solution of 4,4'-bipyridine (2 g, 12.8 mmol) dissolved in 20 mL acetonitrile, 1-bromohexane (0.597 mL, 4.26 mmol) was added. The resultant solution was refluxed at 81 ^{o}C for 24 hrs. After cooling, the yellow precipitate formed was filtered and suspended in hot DMF. The mono alkylated product got dissolved in DMF, while the yellow insoluble di-alkylated product was removed by filtration. Ether was then added to the cooled solution of DMF to precipitate the pale-yellow mono-alkylated salt. Yield = 32% (0.8826 g).

^{1}H NMR (500 MHz, CD_3OD), δ (ppm): 9.032 (d, *J* = 5.5 Hz, 2H) 8.755 (d, *J* = 5.5 Hz, 2H), 8.438 (d, *J* = 6.5 Hz, 2H), 7.907 (d, *J* = 4.5 Hz, 2H), 4.607-4.578 (m, 2H), 2-1.955 (m, 2H), 1.360-1.202 (m, 6H), 0.860-0.833 (m, 3H). ^{13}C NMR (125 MHz, CH_3OD), δ (ppm): 154.30, 150.41, 147.32, 142.89, 125.76, 122.16, 61.40, 31.04, 30.90, 25.49, 22.07, 12.86. HRMS (ESI): m/z calcd., for $C_{16}H_{21}N_2$, 241.17 [M]; found, 241.17 $[M]^{+}$.

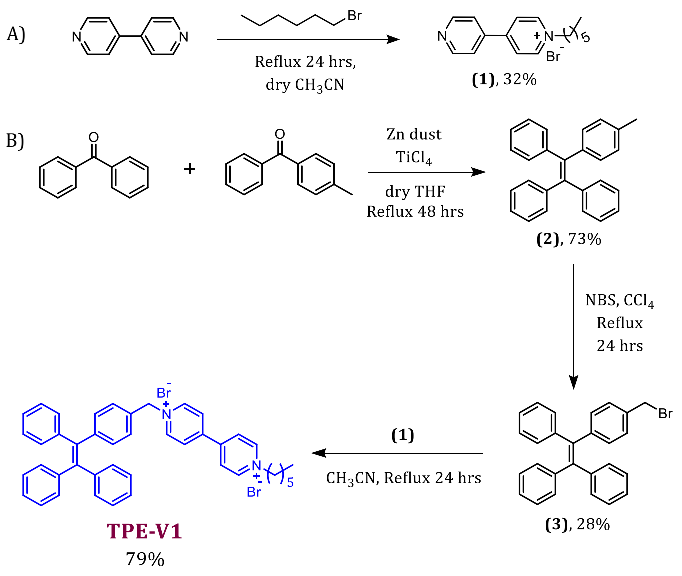

Scheme S1 | Synthesis of mono-viologen functionalized TPE, **TPE-V1**.

2.2. Synthesis of (2-(p-tolyl)ethene-1,1,2-triyl)tribenzene, (2) (Ma et al., 2018)

Into a 250 ml three necked RB flask, 4-methylbenzophenone (3.0116 g, 15.2 mmol), benzophenone (2.8191 g, 15.2 mmol) and Zn dust (8.906 g, 136 mmol) were added. The flask was evacuated and flushed with argon 3 times. After the addition of dry THF (150 mL), reaction mixture was cooled in ice bath to 0 ^{o}C and $TiCl_4$ (7.5 mL, 68.4 mmol) was slowly injected. The mixture was stirred at room temperature for 30 minutes and then refluxed under argon condition for 48 hrs. The mixture was then extracted with ethyl acetate and organic part was dried over anhydrous Na_2SO_4. The crude product was purified by silica column chromatography using hexane as eluent to give a white solid. Yield = 73% (3.8796 g).

^{1}H NMR (500 MHz, $CDCl_3$), δ (ppm): 7.90 (s, 9H), 7.03-7.01 (m, 6H), 6.91-6.88 (m, 4H), 2.25 (s, 3H). ^{13}C NMR (125 MHz, $CDCl_3$), δ (ppm): 143.72, 140.92, 140.72, 140.39, 136.05, 131.34, 128.37, 127.64, 126.4, 21.18. HRMS (ESI): m/z calcd., for $C_{27}H_{22}$, 346.46 [M]; found 346.17 $[M]^+$.

2.3. Synthesis of (2-(4-(bromomethyl)phenyl)ethene-1,1,2-triyl)tribenzene, (3)

1 (1.5825 g, 4.4 mmol), freshly recrystallized NBS (0.9936 g, 5.1 mmol) and catalytic amount of BPO (0.015 g) were taken in a 100 mL RB flask and 60 mL of CCL_4 was added. The solution was refluxed for 24 hours before it was cooled to room temperature. The precipitate was filtered and washed with dichloromethane. The filtrate was extracted with dichloromethane, and the organic fraction was dried under reduced pressure. The crude product thus obtained was further purified with silica gel (230-400 mesh) column chromatography using 2% ethyl acetate-hexane as eluent to yield a white solid. Yield = 28% (0.5381 g).

^{1}H NMR (500 MHz, $CDCl_3$), δ (ppm): 7.107-7.105 (m, 11H), 7.018-7.011 (m, 8H), 4.414 (s, 2H). ^{13}C NMR (125 MHz, $CDCl_3$), δ (ppm): 131.68, 131.34, 131.30, 128.42, 127.93, 127.75, 127.73, 127.66, 126.55, 33.65. HRMS (ESI): m/z calcd., for $C_{27}H_{21}Br$, 425.36 [M]; found 426.36 $[M+1]^+$.

2.4. Synthesis of [1-hexyl-1'-(4-(1,2,2-triphenylvinyl)phenyl)-(4,4'-bipyridine)-1,1'-diium] dibromide, (TPE-V1)

To a solution of **3** (0.538 g, 1.26 mmol) in acetonitrile (20 mL), **1** (0. 3059 g, 1.26 mmol) was added and refluxed at 84 ^{o}C for 24 hrs. After cooling to room temperature, precipitated product was filtered, washed with acetonitrile and dried to give the product as pale-yellow powder. Yield = 79% (0.59 g).

^{1}H NMR (500 MHz, DMSO-d_6), δ (ppm): 9.412-9.355 (m, 4H), 8.755-8.724 (m, 4H), 7.325 (d, *J* = 8 Hz, 2H), 7.135-7.106 (m, 9H), 7.061 (d, *J* = 8 Hz, 2H), 6.979-6.965 (m, 2H), 6.950-6.936 (m, 4H), 5.834 (s, 2H), 4.681-4.652 (m, 2H), 1.958 (s, 2H), 1.292 (s, 6H), 0.872-0.859 (s, 3H). ^{13}C NMR (125 MHz, DMSO-d6), δ (ppm): 146.16, 143.26, 131.78, 131.56, 131.34, 131.03, 130.97, 128.72, 123.54, 128.40, 128.31, 127.58, 127.19, 71.9, 61.8, 31.14, 31.01, 25.52, 22.32, 14.29. HRMS (ESI): m/z calcd., for $C_{43}H_{42}N_2^{2+}$, 586.33 [M]; found 745.16 $[M+2Br+1]^+$.

2.5. Synthesis of (E)-1,2-diphenyl-1,2-di-p-tolylethene, (4) (Liang et al., 2014)

Reaction follows the same McMurry coupling procedure detailed as in the synthesis of **2**. 4-methylbenzophenone (2.01 g, 10 mmol), Zn dust (6.093 g, 91 mmol), TiCl4 (5.02 mL, 45 mmol) and 120 mL of dry THF were used for this reaction. Using hexane as eluent, the crude product was purified by silica column chromatography to give the product as white solid. Yield = 53% (3.87 g).

^{1}H NMR (500 MHz, $CDCl_3$), δ (ppm): 7.01-6.93 (m, 10H), 6.84-6.81 (m, 8H), 2.19 (d, *J* = 10 Hz, 6H). 13C NMR (125 MHz, $CDCl_3$), δ (ppm): 143.11, 139.87, 134.87, 130.33, 130.14, 127.35, 127.35, 126.57, 125.15, 20.18. HRMS (ESI): m/z calcd., for $C_{28}H_{24}$, 360.19 [M]; found 360.18 $[M]^+$.

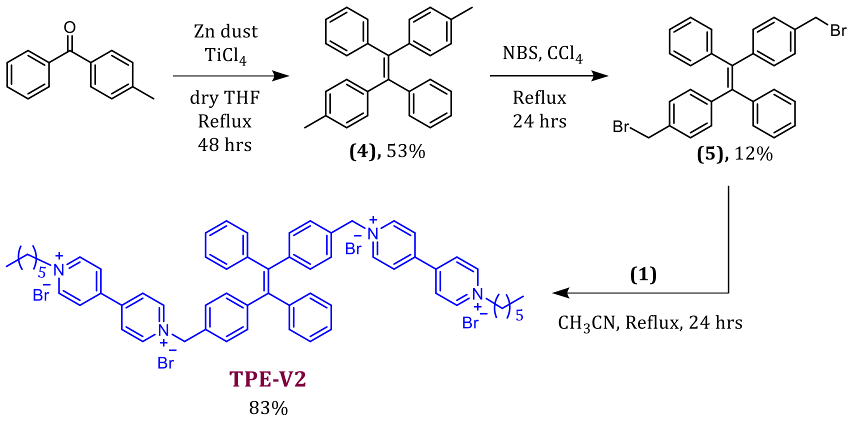

Scheme S2 | Synthesis of di-viologen functionalized TPE, **TPE-V2**.

2.6. Synthesis of (E)-1,2-bis(4-(bromomethyl)phenyl)-1,2-diphenylethene, (5) (Liang et al., 2014)

4 (1.532 g, 4.16 mmol), freshly recrystallized NBS (1.6095 g, 9.1 mmol) and catalytic amount of BPO (0.03 g) in 70 mL of CCl4 were refluxed for 24 hrs. After cooling, the precipitate was filtered and washed with DCM. The filtrate was extracted with DCM and concentrated under reduced pressure. The crude product was then purified using silica gel column (230-400 mesh) with hexane-ethyl acetate as an eluent to yield product as white solid. Yield = 12% (0.260 g).

^{1}H NMR (500 MHz, $CDCl_3$), δ (ppm): 7.108 (m, 10H), 7.000-6.966 (m, 8H), 4.411 (d, *J* = 7 Hz, 4H). ^{13}C NMR (125 MHz, $CDCl_3$), δ (ppm): 143.79, 143.19, 140.79, 135.83, 131.65, 131.31, 128.43, 127.84, 126.75, 33.60. HRMS (ESI): m/z calcd., for $C_{28}H_{22}Br_2$, 516.01 [M]; found 556.98 $[M+K^++1]^+$.

2.7. Synthesis of (E)-1',1'''-(((1,2-diphenylethene-1,2-diyl)bis(4,1- phenylene))bis(methylene))bis(1-hexyl-[4,4'-bipyridine]-1,1'-diium) tetrabromide, (TPE-V2)

A solution of **5** (0.241 g, 0.46 mmol) and **1** (0.168 g, 0.7 mmol) dissolved in dry acetonitrile (15 mL) was refluxed for 24 hrs. After cooling, precipitated product was filtered, washed with acetonitrile and dried to give product as bright yellow solid. Yield = 83% (0.325 g).

^{1}H NMR (500 MHz, DMSO-d_6), δ (ppm): 9.437 (d, *J* = 16 Hz, 8H), 8.799 (s, 8H), 7.348- 7.126 (m, 8H), 7.109-7.033 (m, 3H), 6.979-6.912 (m, 7H), 5.861 (s, 4H), 4.688 (s, 4H), 1.962 (s, 4H), 1.292 (s, 12H), 0.871-0.858 (d, *J* = 6.5 Hz, 6H). ^{13}C NMR (125 MHz, DMSO-d_6), δ (ppm): 149.63, 146.33, 141.21, 140.03, 137.61, 134.32, 132.67, 128.74, 128.41, 128.5, 127.60, 127.19, 71.82, 61.36, 31.17, 31.04, 25.54, 22.34, 14.31. HRMS (ESI): m/z calcd., for $C_{60}H_{64}N_{44}^+$, 840.51 [M]; found 886.79 $[M+2Na^+]^+$.

3. Supporting figures

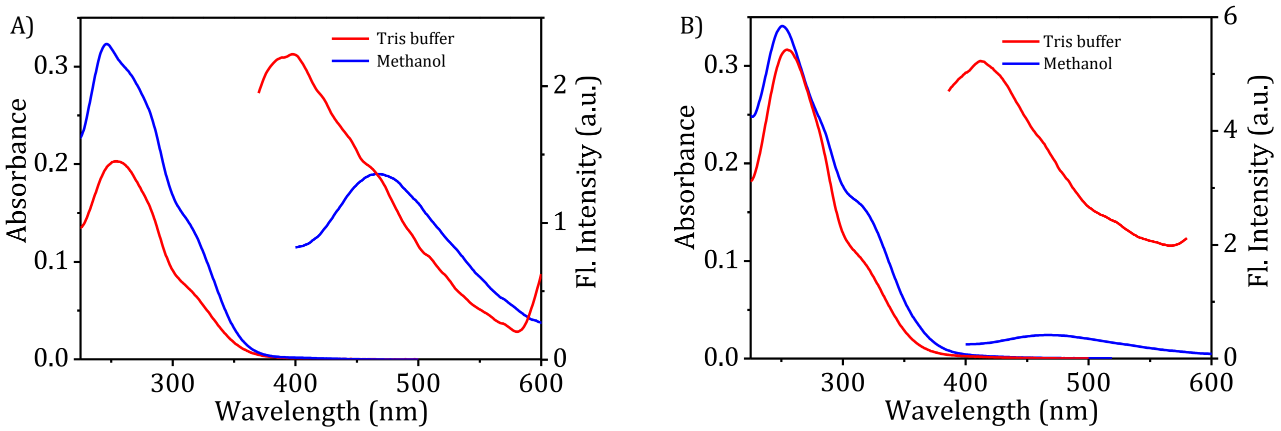

Figure S1 | Absorption and emission spectra (λ_{exc}: 315 nm) of A) **TPE-V1** and B) **TPE-V2** in methanol (blue) and tris buffer (red).

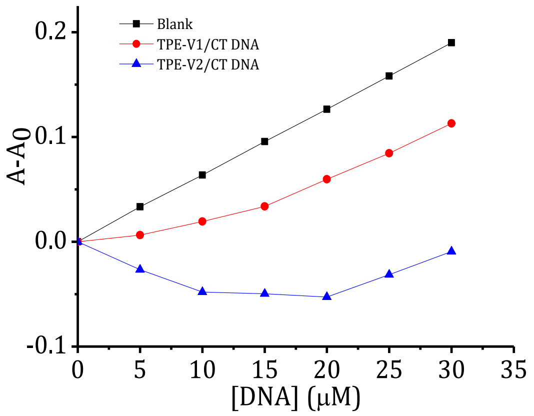

Figure S2 | Changes in the absorption spectra of 15 μM **TPE-V1** and 10 μM **TPE-V2** in buffer system and blank (buffer alone, 10 mM Tris buffer and 2 mM NaCl) at 260 nm as a function of CT-DNA concentration.

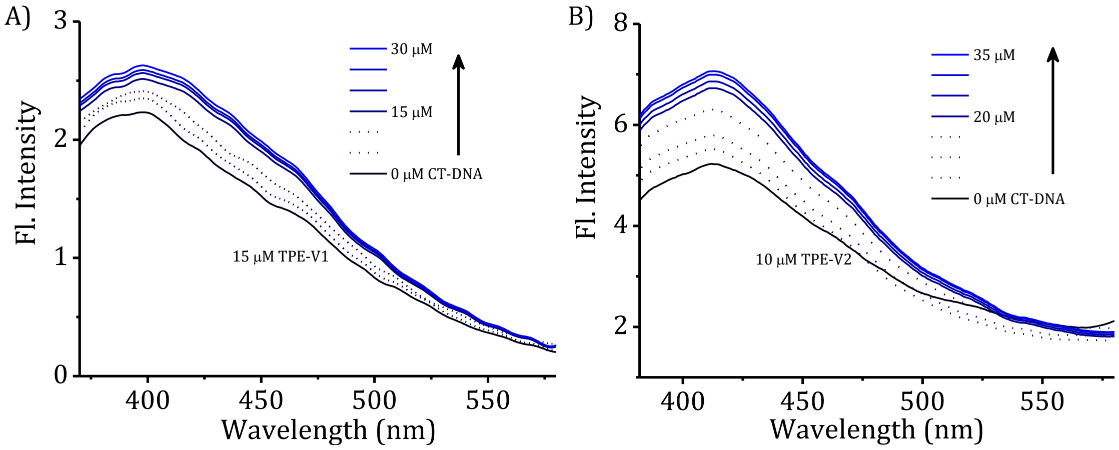

Figure S3 | Changes in the emission spectra of A) **TPE-V1** (15 μM), B) **TPE-V2** (10 μM) in Tris buffer with sequential addition of CT-DNA (each single addition makes 5 μM in the final volume).

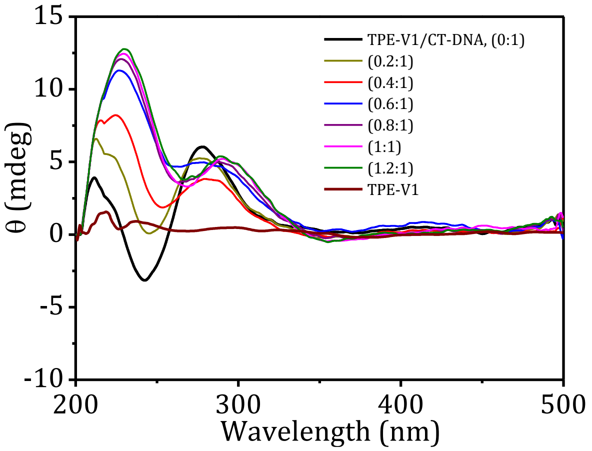

Figure S4 | CD spectra of CT-DNA (100 μM), **TPE-V1** (100 μM) and changes in the CD spectrum of CT-DNA with sequential addition of **TPE-V1** (0 to 1.2 molar equivalent).

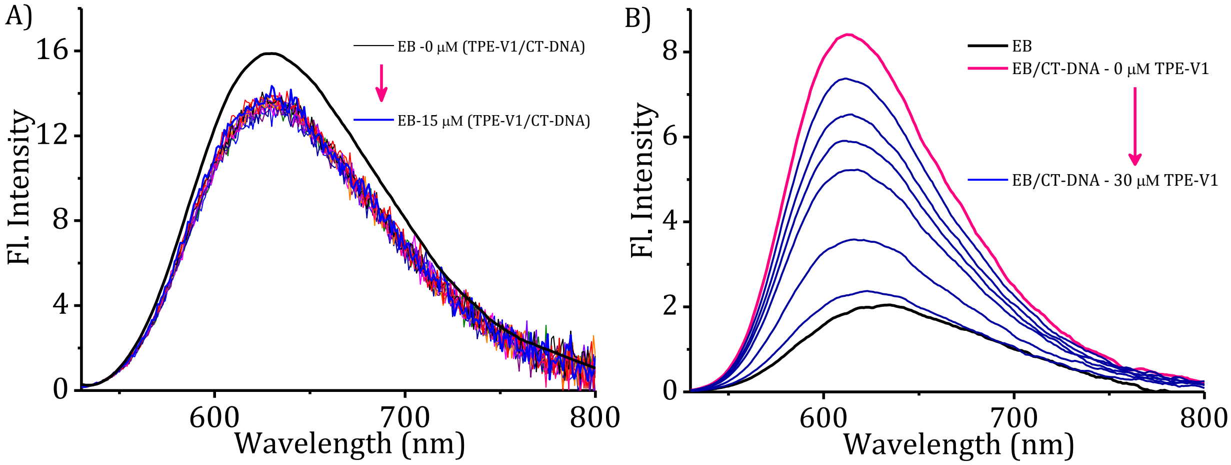

Figure S5 | Changes in the emission spectra of A) EB (15 μM) in presence of increasing concentrations of **TPE-V1/CT-DNA** (1:1 complex, 0-15 μM) and B) EB/CT-DNA complex (1:2) with sequential addition of **TPE-V1** **(**0-30 μM)

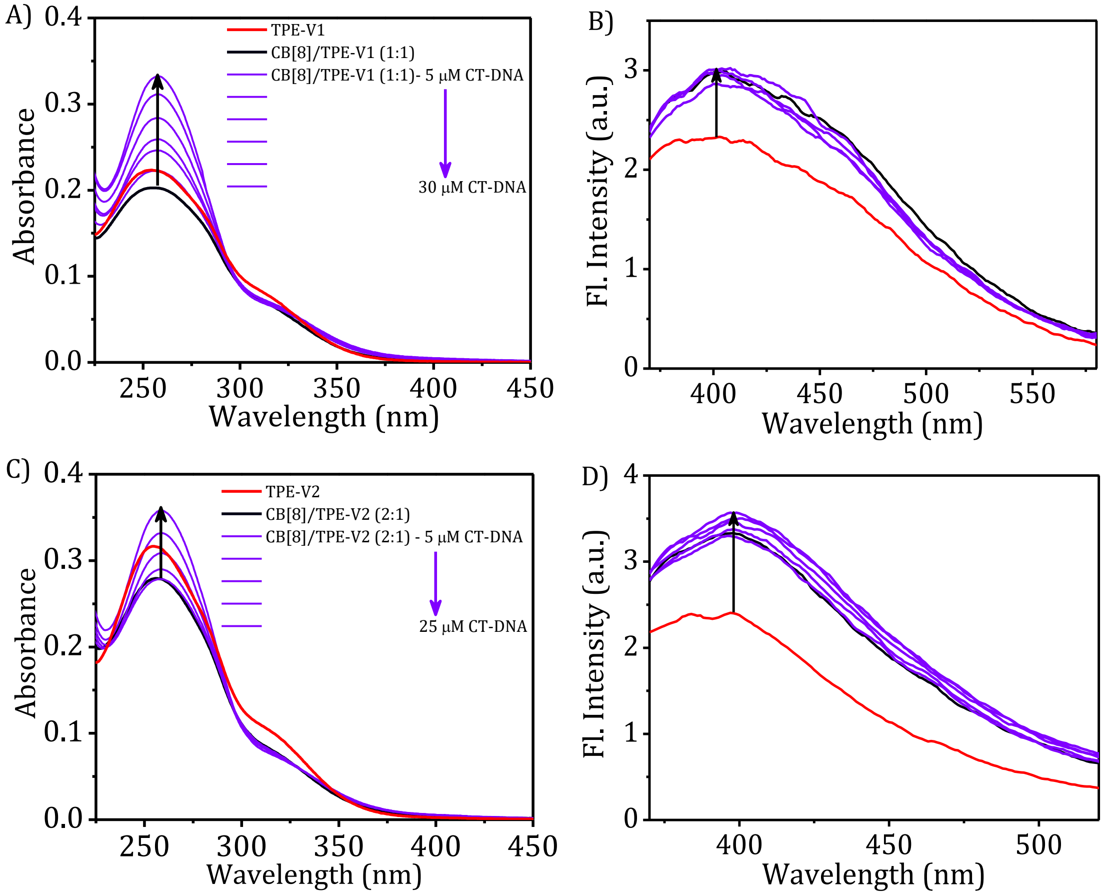

Figure S6 | Changes in the UV-visible absorption and fluorescence emission spectra of A) and B) CB[8]/**TPE-V1** (1:1, 15 μM), and C) and D) CB[8]/**TPE-V2** (2:1, 20 μM:10 μM) with sequential addition of CT-DNA.

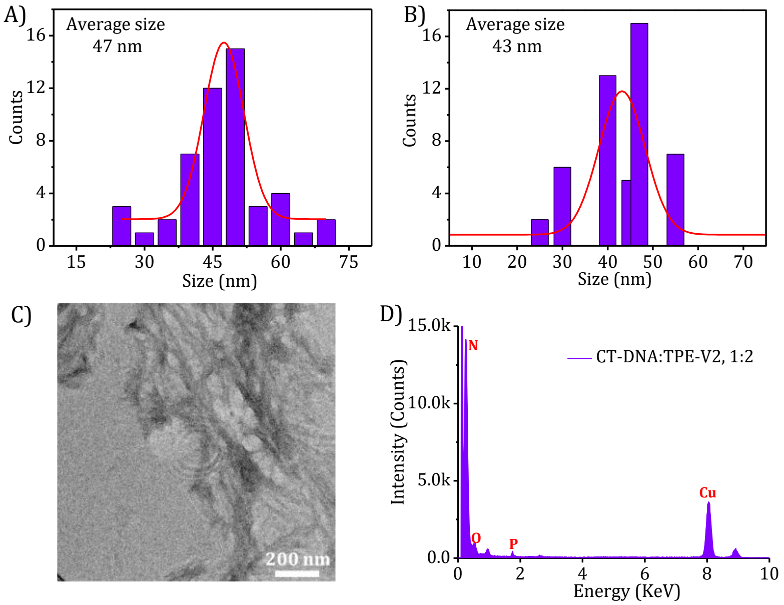

Figure S7 | Histogram plots (Gaussian fit) for the particle size distribution in AFM images A) Figure 4E and B) Figure 5G, C) TEM image of CT-DNA (15 µM) and D) EDAX spectra of **TPE-V2/CT-DNA** complex in 1:2 ratio.

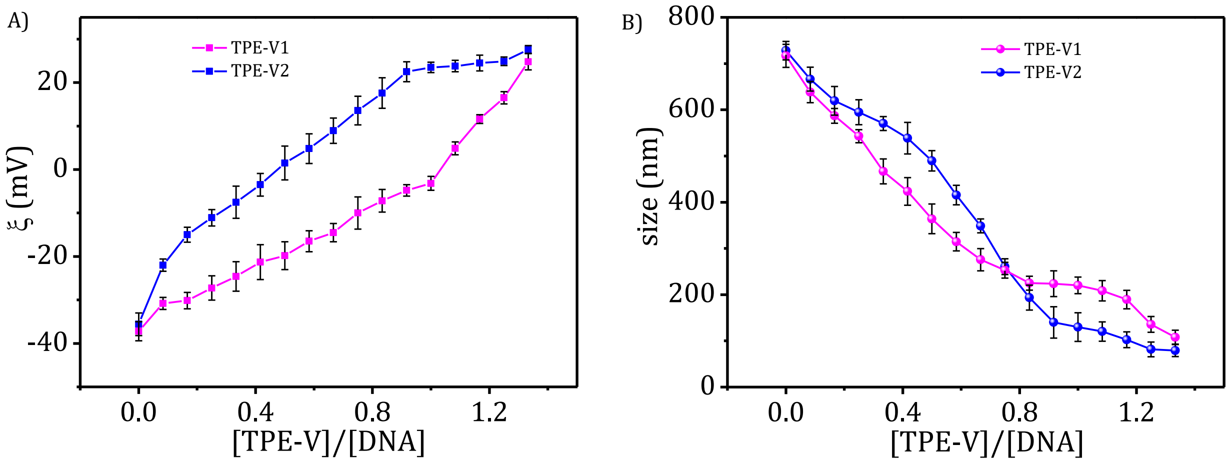

Figure S8 | Changes in the A) zeta-potential and B) hydrodynamic size of CT-DNA (100 µM) with increasing concentrations of **TPE-V**. Each data points represent the mean of three independent experiments (±SD).

4. References

Causin, V., and Saielli, G. (2009). Effect of asymmetric substitution on the mesomorphic behaviour of low-melting viologen salts of bis(trifluoromethanesulfonyl)amide. *J. Mater. Chem.* 19(48)**,** 9153-9162. doi: 10.1039/B915559G.

Liang, J., Shi, H., Kwok, R.T.K., Gao, M., Yuan, Y., Zhang, W., et al. (2014). Distinct optical and kinetic responses from E/Z isomers of caspase probes with aggregation-induced emission characteristics. *J. Mater. Chem. B.* 2(27)**,** 4363-4370. doi: 10.1039/C4TB00405A.

Ma, L., Wang, S., Li, C., Cao, D., Li, T., and Ma, X. (2018). Photo-controlled fluorescence on/off switching of a pseudo[3]rotaxane between an AIE-active pillar[5]arene host and a photochromic bithienylethene guest. *Chem. Commun.* 54(19)**,** 2405-2408. doi: 10.1039/C8CC00213D.
